# Contrasting activity patterns determined by BrdU incorporation in bacterial ribotypes from the Arctic Ocean in winter

**DOI:** 10.3389/fmicb.2013.00118

**Published:** 2013-05-20

**Authors:** Pierre E. Galand, Laura Alonso-Sáez, Stefan Bertilsson, Connie Lovejoy, Emilio O. Casamayor

**Affiliations:** ^1^Biogeodynamics and Biodiversity Group, Centre d’Estudis Avançats de Blanes, Centre d’Estudis Avançats de Blanes-Consejo Superior de Investigaciones CientíficasBlanes, Spain; ^2^UPMC Univ Paris 06France; ^3^CNRS, UMR 8222, Laboratoire d’Ecogéochimie des Environnements Benthiques, Observatoire Océanologique de BanyulsBanyuls sur Mer, France; ^4^Department of Ecology and Genetics, Limnology, Uppsala UniversityUppsala, Sweden; ^5^Centro Oceanográfico de Gijón, Instituto Español de OceanografíaGijón, Spain; ^6^Département de Biologie, Institut de biologie intégrative et des systèmes, Université LavalQC, Canada

**Keywords:** *Colwellia*, bacteria, Arctic Ocean, BrdU, activity

## Abstract

The winter Arctic Ocean is one of the most unexplored marine environments from a microbiological perspective. Heterotrophic bacteria maintain their activity at a baseline level during the extremely low-energy conditions of the winter, but little is known about the specific phylotypes that have the potential to survive and grow in such harsh environment. In this study, we aimed at identifying actively growing ribotypes in winter Arctic Ocean seawater cultures by experimental incubations with the thymidine analog bromodeoxyuridine (BrdU), followed by immunocapturing, terminal restriction fragment length polymorphism fingerprinting, cloning, and sequencing the 16S rRNA gene. We incubated water collected at different months over the Arctic winter and showed that the actively growing bacterial fraction, taking up BrdU, represented only a subset of the total community. Among the BrdU-labeled bacterial taxa we identified the *Flavobacteria* *Polaribacter*, the *Alphaproteobacteria* SAR11, the *Gammaproteobacteria* Arctic 96B-16 cluster and, predominately, members of *Colwellia* spp. Interestingly, *Colwellia* sequences formed three clusters (93 and 97% pairwise 16S rRNA identity) that contributed in contrasting ways to the active communities in the incubations. *Polaribacter*, Arctic 96B-16 and one cluster of *Colwellia* were more abundant in the active community represented by the BrdU-labeled DNA. In contrast, SAR11 and two other *Colwellia* clusters were underrepresented in the BrdU-labeled community compared to total communities. Despite the limitation of the long incubations needed to label slow growing arctic communities, the BrdU approach revealed the potential for active growth in low-energy conditions in some relevant groups of polar bacteria, including *Polaribacter* and Arctic 96B-16. Moreover, under similar incubation conditions, the growth of different *Colwellia* ribotypes varied, suggesting that related clusters of *Colwellia* may have distinct metabolic features.

## INTRODUCTION

The winter in the Arctic Ocean is characterized by an almost complete lack of sunlight and water temperatures below zero degrees Celsius. These extreme conditions are expected to have a large impact on the composition and activity of marine microbes in surface waters down to the nitracline (i.e., the depth interval where nitrate concentration rapidly increase from depleted surface concentrations), as carbon and nutrient availability drastically changes when primary production is reduced or stops during darkness. Indeed, changes in the relative abundance of major bacterial groups over the course of the winter (Alonso-Sáez et al., 2008), as well as in their use of dissolved organic substrates (Nikrad et al., 2012), have been previously reported. Interestingly, despite the harsh environmental conditions, heterotrophic bacterial production is sustained at a baseline level during the entire winter (Garneau et al., 2008), and the single-cell activity of some abundant groups of higher taxonomic rank has been shown to be relatively high (Alonso-Sáez et al., 2008).

Identifying metabolically active ribotypes in low productive marine environments is challenging and information on the specific phylotypes that can actively grow in polar winter waters is scarce. Here, we address this challenge by using Bromodeoxyuridine (BrdU) incubations. Labeling of DNA with BrdU was first used in microbial ecology as an alternative to radioactive thymidine (TdR) to measure bacterial growth (Steward and Azam, 1999). BrdU is an analog of TdR that is incorporated into newly synthetized DNA. This feature was also used to identify the pool of growing bacteria within a community (Borneman, 1999; Urbach et al., 1999) and later coupled to microscopy to identify DNA-synthetizing cells by fluorescence *in situ* hybridization (FISH; Pernthaler et al., 2002; Pernthaler and Pernthaler, 2005). More recently BrdU incubations have been combined with 16S rDNA-based analysis to study the spatial distribution of active communities (Taniguchi and Hamasaki, 2007) or identify active bacteria degrading dissolved organic compounds (Mou et al., 2007).

A significant advantage of the BrdU approach is that this method can potentially show differences in the activity of closely related phylotypes. In previous studies of Arctic waters, a high degree of diversity was found within the most abundant phylogenetic groups of marine bacteria (Kirchman et al., 2010; Lovejoy et al., 2011), implying that many closely related ribotypes can co-exist when conditions are favorable. To what extent the specific metabolic characteristics of such related but ecologically distinct phylotypes differ has not yet been studied extensively. Ecological differences based on spatial and temporal distribution patterns have been described for phylotypes of major marine prokaryotic groups such as unicellular cyanobacteria (Rocap et al., 2003; Johnson et al., 2006) or the SAR11 cluster (Brown et al., 2012). However, differences in the specific activity of related clusters in the environment have seldom been reported. By using the BrdU approach, we aimed at identifying bacterial ribotypes with the capacity to grow in Arctic winter waters, potentially including closely related phylotypes that may constitute ecotypes in this marine environment.

## MATERIALS AND METHODS

### SAMPLING, INCUBATIONS AND BrdU IMMUNOCAPTURE

Water was sampled in the Amundsen Gulf, western Arctic, on November 16th 2007 (Nov), January 10th 2008 (Jan), and February 11th 2008 (Feb) from the base of the nitracline to target a well-defined non-surface water mass also studied by (Alonso-Sáez et al., 2010; **Table 1**). Samples were immediately filtered through a 50 μm mesh, divided between 6 acid-rinsed 10 L polycarbonate bottles and incubated in the dark at 2°C for 30 days with 100 μmol L^-^^1^ BrdU. Bacteria assimilating the thymidine analog BrdU into their newly synthesized DNA can then be identified by immunocapture. This subset of the community will then be considered the actively replicating portion of the community. BrdU incubations are usually short (Pernthaler et al., 2002), but due to the slow growth of bacteria during the Arctic winter, we chose to increase incubation times to ensure sufficient BrdU incorporation (see discussion).

**Table 1:** Position of sampling stations and environmental characteristics of the water used for BrdU incubations.

Station ID	Date	Longitude	Latitude	Depth (m)	Temperature (°C)	Salinity
1117	2007-11-16	126° 30.031 W	69° 52.104 N	20	-0.9	33.8
D14	2008-01-08	125° 35.765 W	71° 31.584 N	80	-1.5	32.3
D19	2008-02-11	124° 47.135 W	71° 4.236 N	50	-1,35	32.6

For the November and January experiments one bottle was sacrificed after 5 days and ca. 10 days. For the February experiment, additional two bottles were sacrificed after 22 and 28 days. Five liters of water were filtered sequentially through a 3 μm pore size polycarbonate filter (Poretics) and a 0.2 μm pore size Durapore filter, using a peristaltic pump. Filters were immediately preserved by freezing at -80°C and DNA was extracted from the 0.2 μm filters as described earlier (Alonso-Sáez et al., 2010). BrdU-labeled DNA (BrdU), representing the active bacterial fraction, was then separated from the total DNA fraction (Tot) by immunocapture. Labeled DNA was captured with anti-BrdU primary antibodies (Mouse IgG monoclonal antibody clone BMC 9318, Roche) followed by isolation using magnetic beads with secondary antibodies (Dynabeads M-450 coated with goat anti-mouse IgG, Invitrogen). DNA was then precipitated with 3M sodium acetate (NaAc) and 99% EtOH. Non-BrdU controls (i.e., seawater cultures without addition of BrdU) were also incubated in the February experiment. For these controls no polymerase chain reaction (PCR) amplification was obtained after the immunocapture procedure. This result, together with the lack of amplification of BrdU labeled DNA after incubation times shorter than 10 days, suggests that no false positives (non-BrdU-labeled DNA) was captured in our study.

### TRFLP, CLONING-SEQUENCING, AND STATISTICAL ANALYSIS

Bacterial 16S rRNA sequences were amplified for terminal restriction fragment length polymorphism (TRFLP) analysis with primers 27f (Vergin et al., 1998) labeled with hexachlorofluorescein (HEX) at the 5 end, and 519r (Lane, 1991) as described earlier (Lymer et al., 2008). Triplicate PCR products were digested using three restriction enzymes in parallel: HaeIII, HhaI, and HinfI. Fragments were separated and detected with an ABI 3730 capillary sequencer together with internal size standards. TRFLP peaks were determined with GeneMarker 1.51 (SoftGenetics) following thresholds and criterions published earlier (Lymer et al., 2008). The different data sets from the three different restriction enzymes were grouped together and a matrix based on peak intensity was built to compare community composition between samples. Jaccard’s similarity coefficient was calculated to compare communities by non-metric multidimensional scaling (NMDS). Analysis of similarity (ANOSIM) statistics were used to verify the significance of the grouping by testing the hypothesis that samples from a same group were more similar in composition with each other than with communities in different group.

In order to taxonomically identify TRFLP peaks we amplified, cloned (TOPO TA kit, Invitrogen) and sequenced bacterial 16S rRNA sequences with primers 27f-1492r from the total DNA fraction for the three incubations. Theoretical terminal restriction fragments were then generated for each enzyme with the program TRiFLe (Junier et al., 2008) with the 159 cloned sequences and with 81 sequences from incubated sea water published earlier from the same region (Alonso-Sáez et al., 2010), and environmental TRFLP peaks were identified by comparison with theoretical TRFLP data. Peak identities were verified with T-RFPred (Fernandez-Guerra et al., 2010) using 6370 sequences (>1000 bp) from the Global Ocean Sampling (GOS) database digested *in silico* with the three enzymes we used, as in (Fernandez-Guerra et al., 2010). Cloned sequences were taxonomically identified using BLAST and best matching sequences were selected to build a phylogenetic tree. The ca. 800–base-pair sequences were aligned using MUSCLE (Edgar, 2004) and manually checked. DNADIST from the program PHYLIP version 3.68 (Felsenstein, 2008) was used to calculate genetic distances with Kimura-2 model and the distance tree was estimated with FITCH. Sequence data have been archived in the GenBank database under accession numbers JN399060-JN399069.

A similarity percentage analysis (SIMPER; Clarke, 1993) was conducted to identify the phylotypes contributing the most to the dissimilarity between BrdU labeled and total community. SIMPER calculates the average Bray-Curtis dissimilarity for all phylotypes between groups of samples and is expressed as average contribution for each phylotype. The significance of the difference between means was then calculated with a Wilcoxon test for paired values. Data analysis were performed with paleontological statistics (PAST) software package (Hammer et al., 2001).

## RESULTS AND DISCUSSION

**FIGURE 1:**
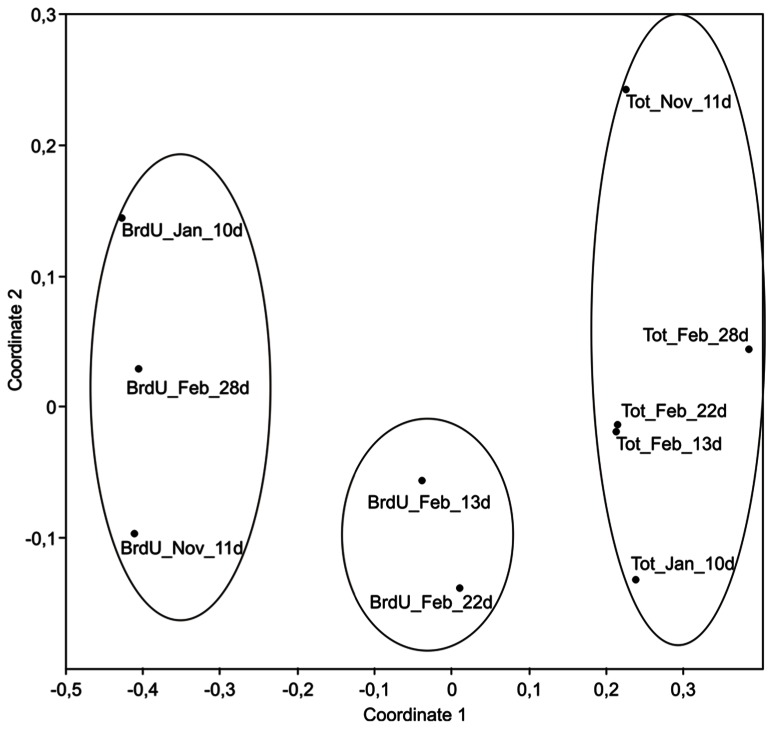
**Non-metric multidimensional scaling (NMDS) plots derived from TRFLP peak intensity data showing similarity between active (BrdU) and total (Tot) bacterial communities**. Date of sampling is indicated by the month: November (Nov), January (Jan), and February (Feb). Length of incubation is shown by the number of days (d). Circles indicate samples grouping at a 60% similarity level. Stress value = 0.09.

BrdU-labeled DNA (BrdU), representing the actively dividing bacterial fraction, was successfully separated from the total DNA fraction (Tot) by immunocapture in incubations of winter Arctic seawater. The BrdU method had been successfully applied in different freshwater and marine environments, but its application in the extremely low productive winter Arctic water was challenging. Therefore, we increased the concentration of BrdU and the incubation time as compared to previous studies. While short incubation times are generally desirable in order to obtain results representative of *in situ* conditions, we found that only BrdU-labeled DNA incubated for at least 10 days was successfully amplified by PCR in the winter Arctic seawater incubations. According to estimates based on the different phases of cell cycles, a BrdU incubation time of 65% of the doubling time would label 100% of all growing cells (Pernthaler and Pernthaler, 2005). In winter waters of the Amundsen Gulf, a prokaryotic growth rate of 0.1 d^-^^1^ was measured in dilution cultures (Alonso-Sáez et al., 2010), which indicates a doubling time of ca. 7 days. Therefore, theoretically, the labeling of actively growing Arctic communities would take ca. 4.5 days. However, growth rates in our incubations were probably lower as we incubated the water without dilution. That may explain that insufficient BrdU-labeled DNA could be extracted from samples collected during the first week of incubation. In the experiment carried out in February, additional BrdU samples were analyzed after 22 and 28 days of incubation. For all the samples, the active and total community DNA was amplified and analyzed by TRFLP (**Figure 1**).

As shown by the TRFLP analysis, BrdU-labeled communities were different from the total communities, and NMDS grouped all labeled samples away from non-labeled samples (**Figure 1**; ANOSIM, *p* < 0.01). This indicated that the actively growing bacterial fraction (BrdU) represented only a subset of the total community. Interestingly, during the February experiment the composition of the total bacterial community changed little from 13 to 28 days of incubation, while differences in the active communities were detected over the course of the experiment, particularly from 22 to 28 days (**Figure 1**). The active bacterial communities after ca. 10 days of incubation in November and January showed higher similarity, but were different to the active community in the experiment of February after 13 days. As incubation conditions were strictly similar for the three experiments, our results suggest that Arctic communities contained different bacterial phylotypes that varied in their potential for growth during the course of the winter.

It should be emphasized that the studied communities do not directly represent *in situ* active bacterial communities, as incubations may promote or limit the growth of some bacteria from the natural assemblages, particularly when long incubation periods are required and high concentrations of potentially inhibiting compounds such as BrdU are applied. Other potential pitfalls in our approach are that the fingerprinting techniques such as TRFLP may miss some bacterial taxa, and that the BrdU substrate could potentially be toxic for some bacteria. For example, early studies have shown that a strain of *Bacillus subtilis* had reduced sporulation and growth under BrdU incubation (Binnie and Coote, 1986). We were, however, able to detect different bacteria in the BrdU fraction, such as SAR11, Polaribacter, Arctic96B -16 and *Colwellia*, which are known to be environmentally relevant in Arctic waters (Bano and Hollibaugh, 2002; Alonso-Sáez et al., 2010). Our results thus show that even though some Arctic microorganisms may have been inhibited, a large diversity of them take up BrdU at the concentration added.

The major components of the bacterial communities were identified by cloning and sequencing bacterial 16S rRNA genes from the three experiments, and we used these data to identify the TRFLP peaks by using the program TRiFLe. We obtained 41, 57, and 61 sequences (ca. 950 bp long) from the libraries Tot_Nov_6d, Tot Jan_10d, and Tot_Feb_13d, respectively. Diversity was low in all three libraries, which were dominated by the genus *Colwellia* that represented >88% of the sequences. *Colwellia* is a psychrophilic marine bacteria, which is well adapted to life in cold environments (Methé et al., 2005). Members of *Colwellia* have been detected in a wide range of marine habitats, including polar regions (Deming and Junge, 2005), and appear to be important to carbon and nutrient cycling in the cold marine environments (Methé et al., 2005). *Colwellia* was also abundant in previous Arctic winter water incubations, particularly in samples taken from the nitracline (i.e., 38% of clones; Alonso-Sáez et al., 2010). Clone libraries from *in situ* samples collected at the same location in winter showed communities dominated by *Gammaproteobacteria* and *Alphaproteobacteria*, a composition that is typical for the Arctic Ocean (Lovejoy et al., 2011). The Arctic 96B cluster and *Polaribacter* were also present *in situ* while *Colwellia* represented 4% of the sequences (Alonso-Sáez et al., 2010).

*Colwellia* were earlier shown to be selectively enriched in North Sea seawater incubations (Eilers et al., 2000) and able to outcompete other bacteria during bottle incubations. Thus, *Colwellia* probably dominated in our experiment because they were able to take advantage of the incubation conditions. However, in contrast to previous studies, it is remarkable that this phylotype was able to grow in winter Arctic waters, as the availability of fresh organic substrates is very low. *Colwellia* species have typically been cultured on heterotrophic media and grow from -1 to 10°C (Huston et al., 2000). An analysis of the genome of the psychrophile *Colwellia* *psychrerythraea* 34H has revealed the potential for this strain to degrade fatty acids and polyamides, and suggests a possible C1 metabolism (Methé et al., 2005). This type of metabolism could be related to the fact that *Colwellia* cells showed active uptake of bicarbonate during incubations in Arctic winter seawater, and may be interpreted as a strategy to deal with starvation under resource depleted conditions (Alonso-Sáez et al., 2010).

**FIGURE 2:**
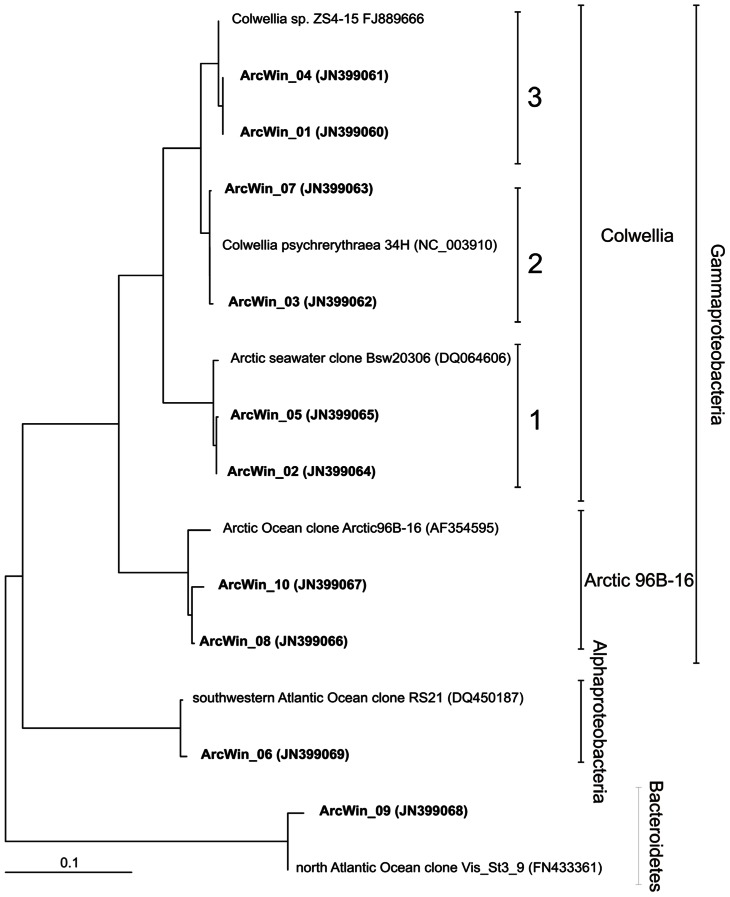
**Distance tree showing the position of bacterial 16S rRNA gene sequences**. Sequences from this study are in boldface type. Analyses are inferred from 16S rRNA gene sequences (length ca. 900 bp) using FITCH distance matrix analysis (from the program PHYLIP). All bootstrap values were >55. Scale bar represents 10% sequence divergence.

*Colwellia* previously isolated from a variety of polar habitats (Bowman et al., 1998; Junge et al., 2002) are very diverse, including the presence of closely related ribotypes. Yet, the ecological significance of their diversity, and the ecological role of different *Colwellia* clusters remain unclear. In our Arctic seawater incubations, *Colwellia* showed intra-genus diversity and sequences fell into three separate clusters (**Figure 2**). Most of the cloned sequences belonged to cluster 3 (123 sequences), represented by the *Colwellia* strain ZS4-15, while fewer fell into cluster 2 and 1 (6 and 12 sequences, respectively). The least abundant cluster 2 was closest to *C.* *psychrerythraea* 34H. Sequences from clusters 2 and 3 had a minimum of 97% identity and sequences from cluster 2 and 1, a minimum of 93% identity. The three *Colwellia* clusters were detected in clone libraries from seawater dilution cultures with Arctic winter waters in an earlier study (11 sequences from cluster1, 1 from cluster 3 and 1 from cluster 2, Alonso-Sáez et al., 2010), and were successfully resolved based on their TRFLP profiles using the software TRiFle

Additional analyses identified other abundant TRFLP peaks, including the *Gammaproteobacteria* Arctic 96B-16 cluster, *Polaribacter*, and SAR11 (**Figure 2**). *Colwellia* cluster 1, *Polaribacter*, and Arctic 96B-16 were more abundant in the active community represented by the BrdU-labeled DNA, indicative of a high potential for growth in Arctic winter waters. In contrast, *Colwellia* cluster 3, SAR11, and *Colwellia* cluster 2 were underrepresented in the BrdU-labeled community compared to total communities (significant difference between means between total and active communities (*p* < 0.05) as calculated with a Wilcoxon test for paired values). Our observation that *Polaribacter* was more frequently found in the active fraction is in agreement with a recent *in situ* study which found that *Polaribacter* had higher leucine uptake rates during winter compared to summer in the Beaufort Sea (Nikrad et al., 2012). It should be noted that other microorganisms present in the incubations might have been overlooked by the TRFP fingerprinting techniques.

**FIGURE 3:**
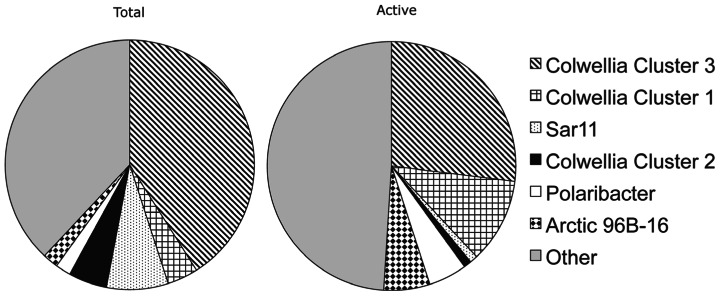
**Proportion of bacterial groups present in the total and the active (BrdU-labeled) fraction of the community**. Data obtained by TRFLP represent averages across all three experiments. *Colwellia* cluster3, cluster1, and SAR11 had significant difference between means between total and active communities (*p* < 0.05) as calculated with a Wilcoxon test for paired values.

The abundant *Colwellia* clusters 3 and 1 showed marked differences in activity in the experiments based on BrdU labeling (**Figure 3**). A SIMPER analysis conducted on TRFLP peaks confirmed that *Colwellia* cluster 1 contributed more than cluster 3 to the active communities across all incubations (**Table 2**). A comparison between months showed that the November and January BrdU samples (BrdU_Nov_11d and BrdU_Jan_10d) contained more *Colwellia* cluster 1 sequences compared to the February sample (BrdU_Feb_13d), characterized by a higher contribution of *Colwellia* cluster 3 (SIMPER analysis, not shown). Such results are consistent with different activity among clusters, but could also reflect differential ability to assimilate BrdU. BrdU uptake capacity and efficiency varies among bacterial genera (Hellman et al., 2011) and could also differ within a single genus, given the high intra-genus diversity of marine microbes. Overall, we conclude that related phylotypes of *Colwellia* showed marked differences in DNA synthesis under similar incubation conditions, and thus hypothesize that they also have consistent differences in their metabolic traits. While these results do not represent changes of the activity of *Colwellia* *in situ*, our data suggest that the two most abundant *Colwellia* clusters present in the incubations likely represent ecologically distinct groups.

**Table 2:** SIMPER analysis identifying the phylotypes that contributed most to the difference between BrdU labeled and total community.

Phylotypes	Contribution	Cumulative %	Mean abundance BrdU	Mean abundance Total
*Colwellia* cluster 3	4.20	6.61	0.27	0.41*
*Colwellia* cluster 1	1.70	24.62	0.11	0.04*
Sar11	1.23	33	0.01	0.08*
*Colwellia* cluster 2	1.01	39.72	0.01	0.05
Polaribacter	0.73	46.28	0.05	0.02
Arctic 96B-16	0.70	50.76	0.06	0.02**

*Cumulative % represents the added contributions of individual phylotypes to the differences between groups*.

* indicates a significant difference between means.

** Significant difference in February only.

In an environment like the winter Arctic Ocean where the bacterial community grows slowly, longer incubation times are needed to label dividing cells. However, such long incubations may also cause changes in the natural communities and is an inherent limitation with the technique. Despite the likelihood that the incubation contents did not reflect the true natural community, the BrdU approach was useful in detecting environmental relevant Arctic bacterial phylotypes with the potential to grow in Arctic winter waters, and highlighting differences among *Colwellia *clades. These results emphasizes that caution is needed when inferring functional roles of taxonomically defined bacterial groups identified by slowly evolving marker genes such as the 16S rRNA. Activity-based studies remain a valuable complement to gene surveys and may reveal functional coherence and incoherence among populations.

## Conflict of Interest Statement

The authors declare that the research was conducted in the absence of any commercial or financial relationships that could be construed as a potential conflict of interest.
